# Expression of TIGIT in splenic and circulatory T cells from mice acutely infected with *Toxoplasma gondii*

**DOI:** 10.1051/parasite/2021010

**Published:** 2021-02-25

**Authors:** Shuai Wang, Haoran Li, Fuqiang Zhang, Yuankai Jiao, Qing Xie, Zhenchao Zhang, Xiangrui Li

**Affiliations:** 1 Xinxiang Key Laboratory of Pathogenic Biology, School of Basic Medical Sciences, Xinxiang Medical University Xinxiang 453003 Henan PR China; 2 Second Clinical Medical College, Xinxiang Medical University Xinxiang 453003 Henan PR China; 3 MOE Joint International Research Laboratory of Animal Health and Food Safety, College of Veterinary Medicine, Nanjing Agricultural University Nanjing 210095 Jiangsu PR China

**Keywords:** *Toxoplasma gondii*, T cells, TIGIT, CD226

## Abstract

The surface protein TIGIT (T cell immunoglobulin and immunoreceptor tyrosine-based inhibitory motif (ITIM) domain) has been characterized as an important regulator of cell-mediated immune responses in various infections. However, TIGIT expression in immune cells of mice infected with *Toxoplasma gondii* has not been investigated. Here, we detected TIGIT expression and related phenotypes by flow cytometry and real-time PCR in splenic and circulatory T cells of mice infected with the *T. gondii* RH strain. We found that the expression of TIGIT on the surface of CD4^+^ T cells and CD8^+^ T cells from the spleen and peripheral blood mononuclear cells decreased in the early stage, but increased significantly in the late stage of acute *T. gondii* infection in mice. Importantly, TIGIT expression was positively correlated with lesions in the murine spleen. In addition, *T. gondii*-specific TIGIT^+^T_CM_ cells in the spleen were activated and transformed into TIGIT^+^ T_EM_ cells. Hematoxylin and eosin staining of spleen sections and real-time PCR showed that the severity of splenic lesions was positively correlated with the *T. gondii* load. This study demonstrates that acute *T. gondii* infection can regulate the expression of TIGIT in T cells and affect immune cell function.

## Abstract (fr)

La protéine de surface TIGIT a été caractérisée comme un régulateur important des réponses immunitaires à médiation cellulaire dans diverses infections. Cependant, l’expression de TIGIT dans les cellules immunitaires de souris infectées par *Toxoplasma gondii* n’a pas été étudiée. Ici, nous avons détecté l’expression de TIGIT et les phénotypes associés par cytométrie en flux et PCR en temps réel dans les cellules T spléniques et circulatoires de souris infectées par la souche RH de *T. gondii*. Nous avons constaté que l’expression de TIGIT à la surface des cellules T CD4 + et des cellules T CD8 + de la rate et des cellules mononucléées du sang périphérique diminuait au stade précoce, mais augmentait de manière significative au stade avancé de l’infection aiguë à *T. gondii* chez la souris. Surtout, l’expression de TIGIT était positivement corrélée avec les lésions de la rate de la souris. De plus, des cellules TIGIT^+^T_CM_ spécifiques de *T. gondii* dans la rate ont été activées et transformées en cellules T_EM_. La coloration à l’hématoxyline et à l’éosine (H&E) des coupes de rate et la PCR en temps réel ont montré que la gravité des lésions spléniques était positivement corrélée à la charge en *T. gondii.* Cette étude démontre qu’une infection aiguë par *T. gondii* peut réguler à la hausse l’expression de TIGIT dans les cellules T et affecter la fonction des cellules immunitaires.

## Introduction

The obligate intracellular parasitic protozoan *Toxoplasma gondii* (*T. gondii*) infects most warm-blooded animals and seriously threatens the health of human beings and animals [7, 20]. It has been reported that more than one-third of people around the world are infected with *T. gondii*, and its incidence is increasing each year [9, 22]. The transmission of *T. gondii* in humans may result from ingestion of food or water contaminated with oocysts excreted by infected cats or ingestion of raw or undercooked meat containing tissue cysts. Transplacental or vertical transmission from the mother to the fetus occurs when tachyzoites pass through the placenta during pregnancy, or medical intervention (e.g., blood transfusion or organ transplantation) [8]. Toxoplasmosis is often asymptomatic when host immune function is normal. When the immune function of the host is impaired, *T. gondii* will spread widely within the host via blood circulation and repeatedly invades and proliferates within host cells, resulting in damage to multiple organs and even death of the host [16].

Cell-mediated immune responses play important roles in *T. gondii* infection [10, 12]. T cells, NK cells, macrophages, INF-γ, TNF-α and NO are all key components of this protective immune response, among which T cells are particularly critical during *T. gondii* infection [10, 17]. A significant feature of the strong immune response of the host to intracellular pathogens is the rapid proliferation of specific T cells and the secretion of multiple functional cytokines. After *T. gondii* infection, specific T cells and NK cells proliferate, mediate cytotoxicity and produce many cytokines, such as TNF-α and IFN-γ, that play critical roles in anti-*T. gondii* infection [19, 23].

Studies have shown that chronic *T. gondii* infection can cause host T cell exhaustion. High expression of inhibitory receptors such as PD-1, TIM-3 and TIGIT [T cell immunoglobulin and immunoreceptor tyrosine-based inhibitory motif (ITIM) domain] on the surface of exhausted T cells significantly inhibited the effector function of T cells [2, 3]. A study found that the proliferative activity of T cells and their ability to secrete factors were restored in a mouse model of *T. gondii* infection after blocking the PD-1-PDL-1 pathway, and the survival rate of the mice increased to 90%. Therefore, it is necessary to identify other checkpoint receptors that mediate T cell exhaustion caused by *T. gondii* infection [3].

TIGIT is encoded on human chromosome 16 and is mainly expressed on NK cells, Treg cells and helper T cells [4, 13]. TIGIT binds to the ligands CD155 (PVR) and CD112 (PVRL2, nectin-2). Both ligands are expressed on antigen presenting cells (APCs) and many non-hematopoietic cell types, including tumor cells, and share a signaling network with the costimulatory receptor CD226 (DNAM-1) [5, 6]. The binding affinity of CD226 for these ligands is approximately 10 times weaker than that of TIGIT. TIGIT can inhibit the interaction between CD226 and CD155 in a dose-dependent manner. In addition to ligand competition, TIGIT can also directly bind to cis isomers of CD226 and disrupt its costimulatory function [11, 15]. Studies have shown that the expression of TIGIT on T cells increased after infection with *Echinococcus multilocularis*, *Plasmodium berghei* or *Schistosoma japonicum*, and was negatively correlated with the immune function of T cells [14, 24, 25]. Nevertheless, it has not been reported whether TIGIT affects the T cell immune response during acute *T. gondii* infection.

In the present study, we aimed to investigate the expression of TIGIT on splenic and circulatory lymphocyte populations and related phenotypes of these cells during acute *T. gondii* infection.

## Materials and methods

### Mice and parasites

The RH strain (type I, virulent strain) of *T. gondii* used in this experiment was preserved by the Department of Human Parasitology, Xinxiang Medical University. Male C57BL/6 mice (7–8 weeks old) were purchased from Beijing Vital River Experimental Animal Technology Co., Ltd., and maintained in animal facility under specific pathogen-free conditions. All animal experiments were reviewed and approved by the Ethics Committee of Xinxiang Medical University (ref. No. 20170305).

### Infection experiments

C57BL/6 mice were divided into an infection group (*n* = 75) and a control group (*n* = 75). The tachyzoites of the RH strain were obtained from the peritoneal fluid of C57BL/6 mice that had previously been inoculated with the RH strain. Mice in the infection group were injected intraperitoneally with 200 tachyzoites of the RH strain, and the same volume of phosphate buffered saline (PBS) solution was injected into the control group.

### Separation and preservation of tissue

Ten mice in each group were randomly sacrificed at 0, 1, 3, 5 and 7 days after infection. Anticoagulated blood was collected aseptically from the mouse orbit, and the red blood cells were removed with erythrocyte lytic solution and then frozen at −80 °C for future use. The spleens of five mice were removed aseptically, ground with liquid nitrogen and frozen at −80 °C for future use. For the other five mice, mouse spleens were removed and immediately fixed in 10% formalin, and 5-μm-thick spleen sections from each mouse were stained with hematoxylin and eosin (H&E) and evaluated for histopathological changes.

### Preparation of peripheral blood mononuclear cells (PBMCs) and splenic mononuclear cells (SMCs)

Five mice in each group were randomly sacrificed at 0, 1, 3, 5 and 7 days after infection. Peripheral blood mononuclear cell (PBMC) isolation was performed by aseptic orbital acquisition of fresh ethylenediaminetetraacetic acid (EDTA) anticoagulant peripheral blood, which was then diluted with PBS preheated to 37 °C. Then, the anticoagulant was slowly spread on the upper layer of lymphocyte separation solution that was preheated to 37 °C and centrifuged at 500× *g* for 20 min; subsequently, different layers were observed. The middle layer containing lymphocytes was adsorbed and transferred to a 15 mL centrifuge tube preloaded with 10 mL of PBS. The sample was centrifuged for 10 min at 4 °C and 400× *g*, and the supernatant was removed. The cell precipitate was resuspended in flow cytometry staining (FACS) buffer (PBS with 2% FCS), and the cells were counted.

Splenic mononuclear cell (SMC) isolation included aseptic isolation of the mouse spleen. Once removed, the spleen was placed in a Petri dish with a diameter of 6 cm (preloaded with 200 mesh/25.4 mm^2^ nylon mesh), and 5 mL of FACS buffer was added. Next, the spleen was cut, and the filter cloth was tightened (the filter cloth was half submerged in PBS). A 20 mL syringe piston was used to grind the spleen until there were no obvious masses, and the cell suspension was collected in a 15 mL centrifuge tube prefilled with 5 mL of 37 °C lymphocyte separation solution. Then, the samples were centrifuged at 500× *g* and 25 °C for 20 min, followed by the same steps described for peripheral blood.

### Flow cytometry

The SMCs and PBMCs were incubated with FcR Blocking Reagent (Miltenyi Biotec, 130-092-575) at 4 °C for 10 min to block non-specific immunoglobulin binding to Fc receptors, stained with Viobility 405/520 Fixable Dye (Miltenyi Biotec, 130-092-575) at 4 °C for 30 min, and washed once with FACS buffer. The cells were incubated with specific antibodies or isotype controls, according to the manufacturer’s guidelines. The antibodies used were as follows: anti-mouse Abs against CD3ε-APC-Vio770, mouse (Miltenyi Biotec, 130-117-676), CD8a-PE-Vio770, mouse (Miltenyi Biotec, 130-102-358), PE/Dazzle™ 594 anti-mouse CD4 Antibody (BioLegend, 100456), Brilliant Violet 421™ anti-mouse TIGIT (Vstm3) Antibody (BioLegend, 142111), Brilliant Violet 421™ Mouse IgG1, κ Isotype Ctrl Antibody (BioLegend, 400157), FITC anti-mouse CD226 (DNAM-1) (BioLegend, 128803), FITC Rat IgG2b, κ Isotype Ctrl Antibody (BioLegend, 400634), PE anti-mouse/human CD44 (BioLegend, 103007), and Alexa Fluor^®^ 488 anti-mouse CD62L Antibody (BioLegend, 104420). All flow cytometry acquisitions were performed using CytoFLEX (Beckman Coulter, Brea, CA, USA) under the same application settings. Flow cytometry data analysis was performed using CytExpert 2.1 software.

### Quantitative real-time PCR

Total RNA was extracted from PBMCs and spleen tissues using TRIzol reagent (Yi Fei Xue Biotechnology, Nanjing, PR China) and then converted to first-strand cDNA using a Script 1st Strand cDNA Synthesis Kit (Yi Fei Xue Biotechnology, Nanjing, PR China), according to the manufacturer’s protocol. cDNA was obtained by reverse transcription PCR on a thermal cycler (Eppendorf, Hamburg, Germany). The product was directly used for quantitative real-time PCR (RT-PCR).

As a measure of parasite load, the *T. gondii* tachyzoite-specific gene SAG1 (TgSAG1) was amplified by RT-PCR [3]. To determine mRNA expression levels of TIGIT and CD226 in the PBMCs and spleens from mice, RT-PCR was performed using ChamQ Universal SYBR qPCR Master Mix (Vazyme Biotech, Nanjing, PR China), and QuantStudio^TM^ 5 (Applied Biosystems, Foster City, CA, USA) was used to analyze the amplification products. The PCR primers used herein are listed in Table 1. The reaction conditions were as follows: initial denaturation at 95 °C for 30 s, followed by 40 amplification cycles (denaturation at 95 °C for 10 s, annealing at 60 °C for 30 s, and extension at 72 °C for 30 s). Values are means from triplicate measurements; specific mRNA expression levels were normalized to the housekeeping gene β-actin mRNA. The results were calculated using the 2^−△△Ct^ method.

**Table 1:** Sequences of the primers used in this study.

GenBank accession	Gene and primer sequence		Target gene length
XM_002368164.2	*T. gondii* SAG1 (TgSAG1)		
	Sense primer	5′–ATCGCCTGAGAAGCATCACTG–3′	101 bp
	Antisense primer	5′–CGAAAATGGAAACGTGACTGG–3′	
NM_007393.5	β-actin		
	Sense primer	5′–GATGCAGAAGGAGATTACTG–3′	91 bp
	Antisense primer	5′–ACCGATCCACACAGAGTA–3′	
NM_001146325.1	TIGIT		
	Sense primer	5′–GGCATGTCGCTTCAGTCTTC–3′	139 bp
	Antisense primer	5′–CTCCCCTTGTAAATCCCACC–3′	
NM_178687.2	CD226		
	Sense primer	5′–ACCACATGGCTTTCTTGCTC–3′	112 bp
	Antisense primer	5′–CAGCATGAGAGTTGGACCAG–3′	

### Statistical analysis

Statistical analysis was performed using SPSS 20 software for Windows (SPSS Inc., Chicago, IL, USA). Student’s *t* test was used to compare the differences between the two groups, and one-way ANOVA was used to compare the differences between multiple groups (one-way ANOVA). The results were considered significantly different when *p* < 0.05.

## Results

### Acute *T. gondii* infection specifically regulated the expression of TIGIT on T cells

**Fig. 1:**
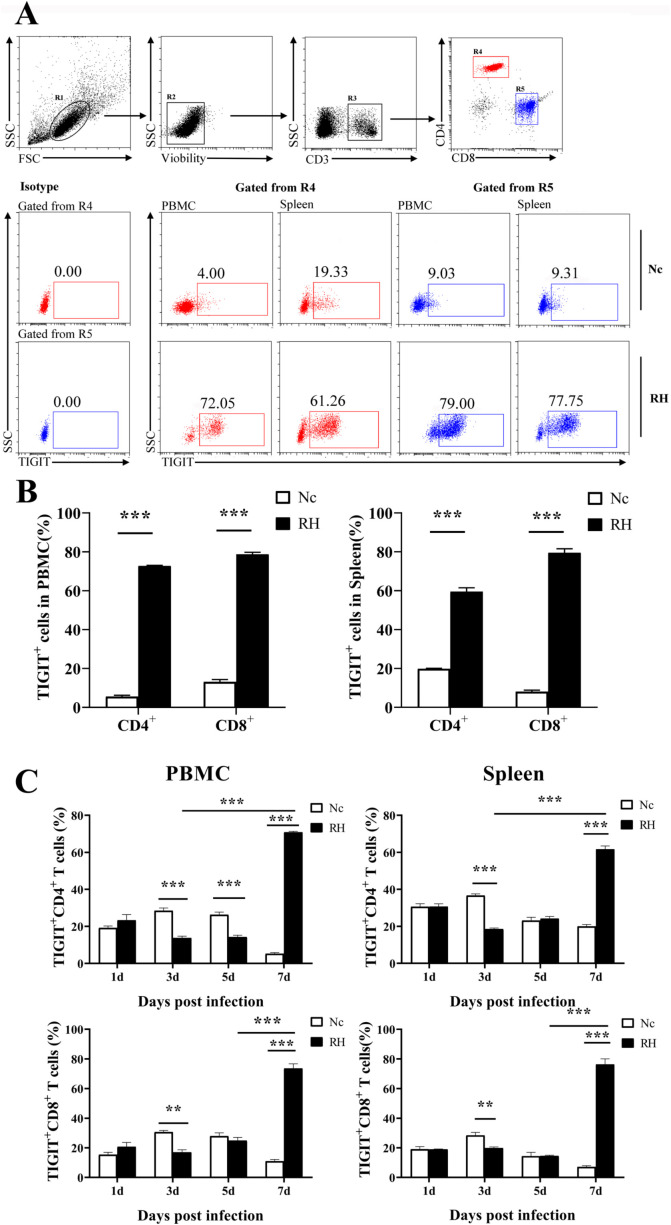
Changes in TIGIT expression on T cells in different tissues after *T. gondii* infection. (A) Proportions of TIGIT^+^ cells among CD4^+^ and CD8^+^ T cells in *T. gondii-*infected (RH) and normal mice (Nc) at 7 days after infection. (B) Results of statistical analysis of the percentage of TIGIT^+^ cells among CD4^+^T and CD8^+^T cells in RH and Nc mice at 7 days after infection. (C) Dynamic changes in the percentages of TIGIT^+^ in T cells at different time points. The results are representative of three independent experiments with five mice in each group per experiment, with data denoting means ± SDs.

Compared with that of the control group, the TIGIT expression of CD4^+^ and CD8^+^T cells among PBMCs of the infection group was significantly upregulated on the 7th day post infection (TIGIT^+^CD4^+^T cell: 5.58% ± 1.30% vs. 72.72% ± 0.64%, *p* < 0.001; TIGIT^+^CD8^+^T cells: 13.15% ± 2.32% vs. 78.72% ± 2.06%, *p* < 0.001). Similarly, the proportion of TIGIT^+^T cells in the spleen of infected mice was much higher than that of the control mice (TIGIT^+^CD4^+^T cells: 19.85% ± 0.41% vs. 59.50% ± 2.5%, *p* < 0.001; TIGIT^+^CD8^+^T cells: 8.07% ± 1.19% vs. 79.48% ± 3.40%, *p* < 0.001). During the infection process, TIGIT expression in T cell subsets within PBMCs and spleens from the infection group decreased on the third day post infection; this trend was more obvious in CD4^+^ T cells, but it only lasted until the fifth day after infection in CD4^+^T cells within PBMCs (Fig. 1).

### Acute *T. gondii* infection selectively downregulated the expression of CD226 on T cells

**Fig. 2:**
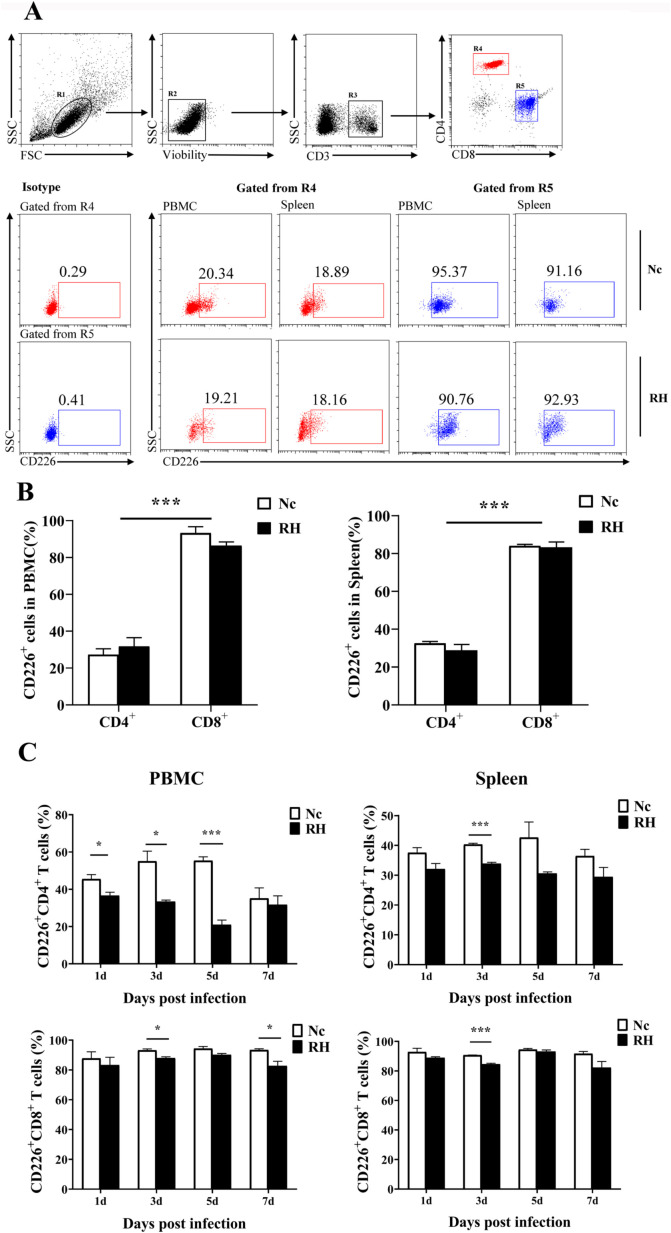
Changes in CD226 expression on T cells in different tissues after *T. gondii* infection. (A) Proportions of CD226^+^ cells among CD4^+^T and CD8^+^T cells in *T. gondii-*infected (RH) and normal mice (Nc) at 7 days after infection. (B) Results of statistical analysis of the percentage of CD226^+^ cells among CD4^+^T and CD8^+^T in RH and Nc mice at 7 days after infection. (C) Dynamic changes in the percentages of CD226^+^ T cells at different time points. The results are representative of three independent experiments with five mice in each group per experiment, with data denoting means ± SDs.

In contrast to that of TIGIT, even at 7 days after infection, the expression of CD226 on T cells was hardly affected by *T. gondii* infection (CD226^+^CD4^+^ T cells among PBMCs: 27.31% ± 5.671% vs. 31.74% ± 4.430%, *p* = 0.48; CD226^+^CD8^+^ T cells among PBMCs: 93.28% ± 3.949% vs. 86.42% ± 6.86%, *p* = 0.16; CD226^+^CD4^+^ T cells in the spleen: 32.53% ± 3.249% vs. 28.80% ± 3.73%, *p* = 0.31; CD226^+^CD8^+^ T cells in the spleen: 84.03% ± 0.725% vs. 83.31% ± 2.275%, *p* = 0.77). CD226 appeared more frequently on the cell surface of CD8^+^ T cells than CD4^+^ T cells. However, on the third day after infection, the number of CD226^+^ T cells was lower in the infection group than that in the Nc group (Fig. 2).

### Acute *T. gondii* infection triggers an increase in T_EM_ cells among TIGIT^+^T cells

**Fig. 3:**
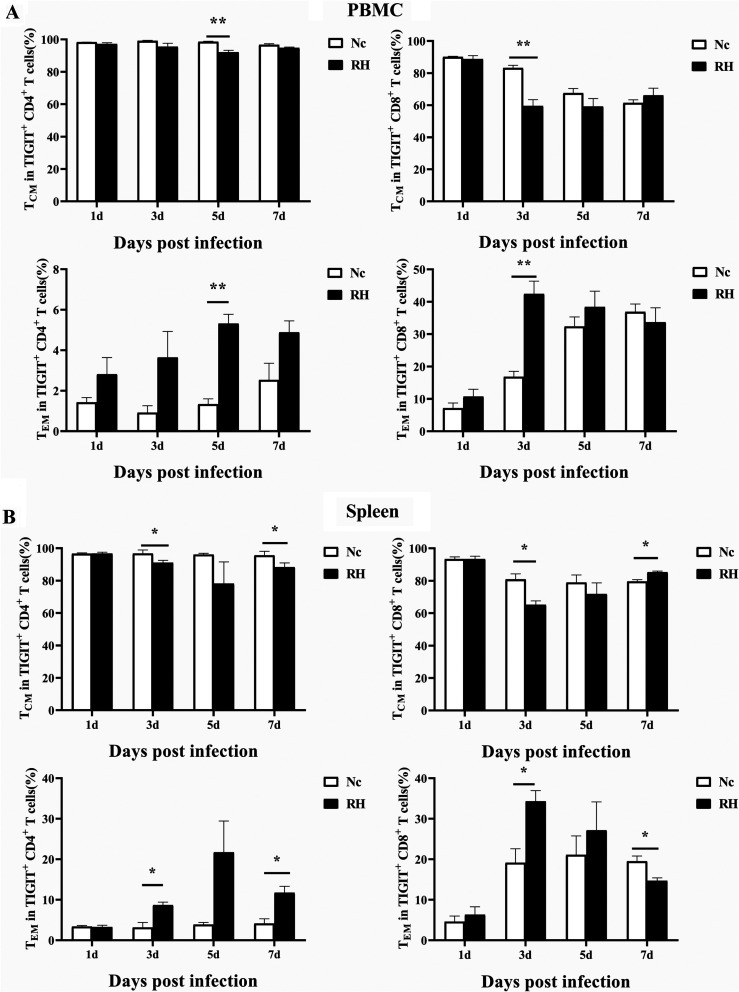
Relative contributions of memory T cell subsets of TIGIT^+^ T cells after *T. gondii* infection. (A) Dynamic changes in memory T cell subsets of TIGIT^+^ T cell in PBMCs at different time points following RH infection. (B) Dynamic changes in memory T cell subsets of TIGIT^+^T cells in the spleen at different time points following RH infection. The results are representative of three independent experiments with five mice in each group per experiment, with data denoting means ± SDs.

Memory T cells play a critical role in providing long-term immunity. Memory T cells are divided into four subsets based on the expression of the cell surface markers CD44 and CD62L. We classified cells based on the expectation that central memory T cells are CD44^hi^CD62L^hi^, effector memory cells are CD44^hi^ CD62L^lo^, effector memory T cells are CD44^lo^CD62L^lo^, and naive memory cells are CD44^lo^CD62L^hi^. Memory T cells subset analysis was performed on both TIGIT^+^CD4^+^ and TIGIT^+^CD8^+^T cells. As shown in Figure 3, we found that T_CM_ and T_EM_ cells were the predominant subsets among *T. gondii*-specific TIGIT^+^T cells. The phenotypic changes of TIGIT^+^T cells in PBMCs were specific. The T_CM_ subset was activated and transformed into T_EM_ cells on the 3rd day after infection in TIGIT^+^CD8^+^ T cells and on the 5th day after infection in TIGIT^+^CD4^+^ T cells. On the 3rd and 7th days post infection, the T_CM_ subset of *T. gondii*-specific TIGIT^+^CD4^+^ T cells in the spleen was activated and transformed into T_EM_ cells. For the TIGIT^+^CD8^+^ T cells in the spleen, the T_CM_ subset was activated and transformed into T_EM_ cells on the 3rd day post infection, but the opposite happened on the 7th day post infection (Fig. 3).

### Histopathological changes in the spleen were aggravated by an increasing *T. gondii* parasite load

**Fig. 4:**
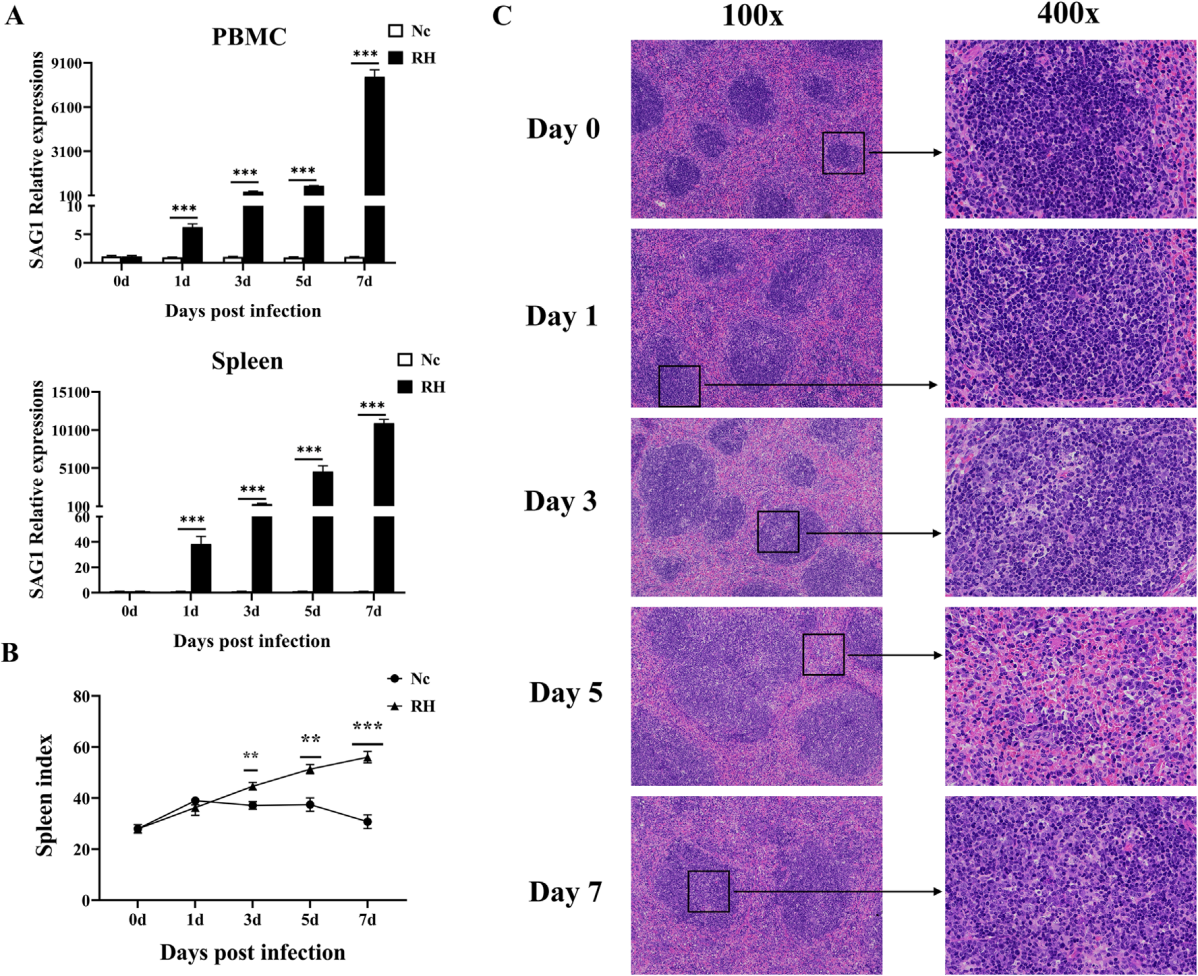
Dynamic pathological changes in the spleen during acute *T. gondii* infection. (A) Relative expression of TgSAG1 at 0, 1, 3, 5, and 7 days post infection in the PBMCs and spleen. (B) Spleen index values of the RH and Nc groups. The results are representative of three independent experiments with five mice per group per experiment; with data denoting means ± SDs; (C) H&E staining of spleen sections at different time points in the RH infection group. The original magnification was 100×, and the corresponding images on the right were magnified at 400×.

The relative expression of the *T. gondii* tachyzoite-specific gene SAG1 in PBMCs and spleen increased significantly from the first day post infection (Fig. 4A). Meanwhile, from the 3rd day after infection, the spleens of mice in the infected group rapidly enlarged, and the spleen index was significantly higher than in the control group (Fig. 4B). Through H&E staining of spleen sections, we observed little change in the spleen on the first day post infection; the splenic sinus was intact, and the course of the splenic cord was normal. On the 3rd day post infection, infiltration of inflammatory cells, congestion in the middle of the splenic cord, and a compensatory increase in lymphocytes were observed. On the 7th day after infection, there was necrosis of spleen cells, complete destruction of the spleen structure, a decrease in lymphocytes, and a large number of tachyzoites and pseudocysts (Fig. 4C). The dynamic changes in TgSAG1 gene expression appeared consistent with the spleen pathology from days 3 to 7 post infection. These results showed that *T. gondii* tachyzoites quickly invaded the circulatory system and were then transported to various organs. Then tachyzoites further proliferated in the spleen by means of an immune escape mechanism and gradually destroyed spleen function.

### TIGIT and CD226 mRNA expression in PBMCs and spleen

**Fig. 5:**
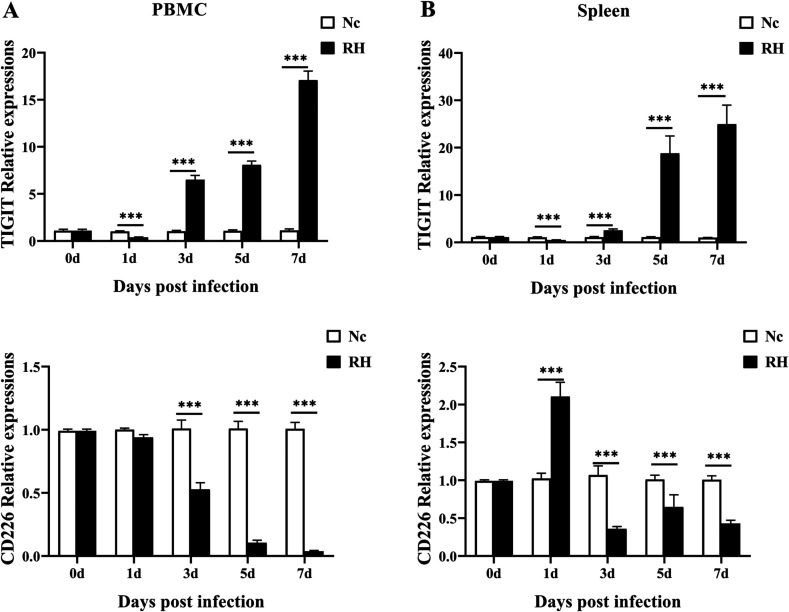
mRNA expression of TIGIT and CD226 in the PBMCs and spleens from mice infected with the *T. gondii* RH strain, as assessed by qRT-PCR. A: Data obtained from PBMCs. B: Data obtained from the spleen. Values are the means from triplicate measurements, with data denoting means ± SDs; three independent experiments were performed with five mice per group. *p < 0.05, **p < 0.01 and ***p < 0.001 (compared to the control).

As shown in Figure 5, the relative expression level of TIGIT downregulated in the PBMCs and spleens of infected mice on the first day after infection, while the expression of costimulatory receptor CD226 in the spleen of infected mice increased significantly. From the 3rd day after infection, the expression of TIGIT in PBMCs and spleens of the infected group was significantly higher than in the control group, and the down-regulation of CD226 was also observed at the same time point. These results showed that with an increasing *T. gondii* parasite load, the increased expression of TIGIT competitively suppressed the expression of CD226, resulting in a significant inhibition of cell-mediated immunity in mice infected with *T. gondii*.

## Discussion

Previous studies have shown that TIGIT plays a pivotal inhibitory role in the immune system. However, research on TIGIT in the context of parasite infection is relatively scarce. Zhang et al. [24] found that the expression of TIGIT, CD3e, CD4 and CD8B was upregulated in the central liver tissue of patients with alveolar echinococcosis, and the expression of TIGIT was positively correlated with the expression of these factors. The same results were also observed in the mouse infection model. Further studies showed that blocking treatment with a TIGIT monoclonal antibody could significantly increase the content of CD4-Teffs (CD4^+^Foxp3^−^ T cells) and the ability of liver infiltrating T cells to secrete cytokines such as IL-2, IFN-γ and TNF-α. Infection with *Plasmodium yoelii* could induce high expression of TIGIT on splenic CD4^+^T cells in infected mice [21]. Zhang et al. [25] further demonstrated that the compensatory increase in TIGIT in *P. berghei* ANKA-infected mice was caused by blockade of the TIM-3-Gal-9 pathway with the TIM-3 ligand Galectin (Gal)-9 blocker α-lactose, which may be one of the causes of death in mice. Zhang et al. [14] found that TIGIT can enhance the proliferation of CD4^+^ T cells in the spleen of mice infected with *S. japonicum* and then induce higher expression of IL-4 and lower expression of IFN-γ to promote activation of the Th2 immune response. However, the expression pattern and function of TIGIT in the immune cells of mice infected with *T. gondii* were unknown.

In this study, we found for the first time that the expression of TIGIT on the surface of CD4^+^ and CD8^+^T cells in the spleen and PBMCs decreased in the early stage but increased significantly in the late stage of acute *T. gondii* infection in a mouse model. On the 3rd day after acute infection, the expression of TIGIT and CD226 decreased in the PBMCs and spleens of the mice, suggesting that the immune system was activated and functional in the early stage of *T. gondii* infection, producing a large number of immune cells, enhancing immune activity, and eliminating free tachyzoites; however, at the same time, *T. gondii* formed pseudocysts to achieve immune escape. In the later stage of infection, *T. gondii* continuously disrupted the immune system, resulting in impaired immune activity. On the 7th day after infection, the expression of TIGIT in PBMCs and the spleen was significantly upregulated, eventually leading to immune failure and death. Studies have shown that the host T cell-mediated immune response plays an important role in the response to pathogen infection. Ackermann et al. [1] found that the number of TIGIT^+^CD4^+^T cells in patients with acute hepatitis C virus (HCV) infection was significantly higher than in healthy controls, and TIGIT was highly expressed in all CD4^+^Tm subsets (memory T cells). However, the mechanism of immune cell failure caused by pathogen infection, as well as whether this process is related to TIGIT, has not been well studied until now.

To further explore the effect of *T. gondii* infection on the phenotypic changes of TIGIT^+^T cells, we analyzed the expression of CD44 and CD62L on TIGIT^+^ T cells by flow cytometry. The results showed that the TIGIT^+^ T cells of both the Nc and RH groups were mainly T_CM_ cells, and the rest were T_EM_ cells. T_CM_ cells usually reside in T cell areas of secondary lymphoid organs and readily proliferate and differentiate into T_EM_ cells, which are the predominant population elicited in response to antigenic stimulation during parasitic infection. These cells have little or no effector function, but persistent antigen stimulation maintains the effector function of memory T cells at a high level, which eventually leads to T cell exhaustion [18]. In this study, *T. gondii*-specific TIGIT^+^ T_EM_ cells in the spleen increased on the 3rd and 7th days post infection, indicating that T_CM_ cells were triggered to differentiate into T_EM_ cells due to the increase in parasite load and antigen stimulation.

In addition, specific TgSAG1 expression in PBMCs and spleen increased significantly after *T. gondii* infection, and the expression of TIGIT was also considerably up-regulated, indicating that *T. gondii* infection can induce a large amount of expression of immunosuppressive receptors in the host, thus activating an immune escape mechanism and massive proliferation in the host, which eventually leads to destruction of the immune system and damage to immune function. Histopathological changes in the spleen were aggravated by an increasing *T. gondii* parasite load. By the 7th day after infection, the splenic structure of infected mice was completely destroyed, and the relative expression of TIGIT increased significantly, while *T. gondii* was still proliferating. These results are consistent with reports that *T. gondii* can cause host T cell exhaustion. However, the effect of TIGIT on the proliferation and cytokine secretion of *T. gondii*-specific T cells is not clear, and whether T cell function can be restored after blocking the TIGIT pathway remains to be studied further.

## Conclusion

Conclusively, our results indicated that acute *T. gondii* infection can increase the expression of TIGIT in host T cells and stimulate the transformation of TIGIT^+^ T_CM_ cells into TIGIT^+^ T_EM_ cells. Whether the increase in TIGIT expression induces exhaustion of host T cells during acute *T. gondii* infection, and whether TIGIT signaling blockade reverses the functional impairment of T cells needs to be studied further.

## Conflict of interest

The authors declare that they have no conflict of interest.
